# An Online Respiratory Quotient-Feedback Strategy of Feeding Yeast Extract for Efficient Arachidonic Acid Production by *Mortierella alpina*

**DOI:** 10.3389/fbioe.2017.00083

**Published:** 2018-01-22

**Authors:** Xiangyu Li, Chao Yu, Jianming Yao, Zhiming Wang, Shuhuan Lu

**Affiliations:** ^1^Hefei Institutes of Physical Science, Chinese Academy of Sciences, Hefei, China; ^2^University of Science and Technology of China, Hefei, China; ^3^CABIO Bioengineering (Wuhan) Co., Ltd, Wuhan, China; ^4^Hubei Province Nutrition Chemicals Biosynthetic Engineering Technology Research Center, Wuhan, China; ^5^Key Laboratory of Systems Bioengineering (Ministry of Education), Tianjin University, Tianjin, China

**Keywords:** *Mortierella alpina*, arachidonic acid, online regulation, feeding strategy, respiratory quotient

## Abstract

*Mortierella alpina* (*M. alpina*) is well known for arachidonic acid (ARA) production. However, low efficiency and unstableness are long existed problems for industrial production of ARA by *M. alpina* due to the lack of online regulations. The aim of the present work is to develop an online-regulation strategy for efficient and stable ARA production in industry. The strategy was developed in 50 L fermenters and then applied in a 200 m^3^ fermenter. Results indicated that yeast extract (YE) highly increased cell growth in shake flask, it was then used in bioreactor fermentation by various feeding strategies. Feeding YE to control respiratory quotient (RQ) at 1.1 during 0–48 h and at 1.5 during 48–160 h, dry cell weight, and ARA titer reached 53.1 and 11.49 g/L in 50 L fermenter, which were increased by 79.4 and 36.9% as compared to that without YE feeding, respectively. Then, the online RQ-feedback strategy was applied in 200 m^3^ bioreactor fermentation and an average ARA titer of 16.82 g/L was obtained from 12 batches, which was 41.0% higher than the control batches. This is the first report on successful application of online RQ-feedback control of YE in ARA production, especially in an industrial scale of 200 m^3^ fermentation. It could be applied to other industrial production of microbial oil by oleaginous microorganisms.

## Introduction

Arachidonic acid (ARA), an omega-6 polyunsaturated fatty acid, is an important component in cell membranes (Barakat et al, 2015). It plays an important role in the development and function of human tissues, immune system, brain growth, and retinal development (Martinez, 1994; Katsuki and Okuda, 1995; Koletzko et al., 2015). Because of its various functions in the physiological activities, ARA has been widely used in the applications of medicine, pharmacology, cosmetics, and food industry, especially in the infant formulas (Dedyukhina et al., 2011; Hadley et al., 2016). Owing to the benefits of ARA, increasing interest had been paid to the production of microbial oil containing ARA for the past decades. *Mortierella* fungi was proved to be an efficient strain for accumulation of ARA-rich oil, thus various fermentation processes had been developed for the improvement of ARA production by *Mortierella* fungi (Ji et al., 2014). Online parameters such as pH and dissolved oxygen are important for ARA industrial production because they could be real-time monitored for regulation of the fermentation process. Li et al. (2015) used a two-stage pH control strategy to get a maximum ARA production of 8.12 g/L, which was 29.3% higher than the natural pH batch in *Mortierella alpina*. Peng et al. (2010a,b) fed 40 g/L of n-hexadecane as an oxygen vector to achieve an ARA titer of 15.9 g/L.

However, the traditional online parameters could not efficiently stabilize the ARA-rich oil production because they could not reflect physiological changes of the cells. Thus, it was necessary to develop an efficient online control strategy for ARA-rich oil production. The biosynthesis pathway of lipid requires large amounts of acetyl-CoA as precursor and NADPH as reducing power (Ratledge, 2004). Acetyl-CoA is primarily produced in the rout of glycolysis and tricarboxylic acid cycle (TCA) and NADPH is generated in the malate/pyruvate cycle with production of CO_2_ and no O_2_ consumed (Wynn and Ratledge, 2000; Wynn et al., 2001; Zhang et al., 2007; Belle and Goossens, 2011). The different fluxes of carbon source into the metabolic network would lead to a change of the quotient of CO_2_ produced and O_2_ consumed, also known as respiratory quotient (RQ). RQ can be easily calculated and recorded online based on O_2_ and CO_2_ content in the outlet gas measured by gas analyzer. Therefore, RQ could be taken as a new parameter to regulate the nutrition feeding. It was widely applied to optimization and scale-up in many fermentation processes, such as erythromycin and glutathione production (Xiong et al., 2010; Chen et al., 2015). Carbon source (Chen et al., 2015) and aeration rate (Guo et al., 2016) were the mainly studied factors in correlation with RQ. However, hardly any attention was paid on the relationship of RQ and the nitrogen feeding. Nitrogen was important for lipid production because lipid accumulation was generally induced in a nitrogen-limitation condition (Belle and Goossens, 2011). Meanwhile, appropriate nitrogen concentration would enhance cell growth, which was also important because lipid synthesis in *M. alpina* was a growth-coupled process (Eroshin et al., 2000). Over feeding or inadequate feeding of nitrogen would negatively affect lipid production and thereby affect ARA titer. Thus, it was necessary to establish an efficient method for the nitrogen feeding. The online RQ might be a new tool to regulate the feeding of nitrogen for ARA production by *M. alpina*.

In this paper, yeast extract (YE) was found to be favorable for cell growth in shake flask culture. The effect of YE feeding on the change of RQ was then studied in 50 L fermenter. Based on the relationship of RQ and YE, three different YE feeding strategies were designed and tested in 50 L fermenters to study how RQ affected the cell growth, lipid and ARA synthesis. Finally, the optimal online-RQ feedback strategy for feeding YE was applied in 200 m^3^ fermenter for efficient and stable ARA production. To our knowledge, this was the first report on the successful application of online RQ-feedback control of YE feeding in ARA production, especially in the industrial scale of 200 m^3^ fermentation.

## Materials and Methods

### Microorganism

*Mortierella alpina* I49-N18, screened from the mutagenesis by ion implantation of *M. alpina* N7 (Yao et al., 2000).

### Culture Condition in Shake Flasks

Slant medium (g/L): potato 200, glucose 25, agar 20.
Seed medium (g/L): yeast powder (YP) (N content: 8.4%, Brewer’s yeast, Angel Yeast Co., Ltd., China) 10, glucose 40, KH_2_PO_4_ 2, sodium glutamate (N content: 8.27%) 5, antifoam agent 0.02, pH 6.0.
Fermentation medium (g/L): YP 15, glucose 80, KH_2_PO_4_ 4, MgSO_4_⋅7H_2_O 0.1, NaNO_3_ 3, pH 6.0.

Stock cultures were propagated on potato dextrose agar slants at 27–29°C for 7–9 days. Spores were harvested and then dispersed in sterile physiological saline, 5 mL spore solution (10^8^/mL) was inoculated into 1,000 mL baffled flask containing 200 mL seed medium and cultivated at 28°C for 48 h under constant orbital shaking at 120 rpm. 20 mL of the seed culture was inoculated into the 1,000 mL shake flask containing 180 mL of the fermentation medium and cultivated at 28°C for 10 days under constant orbital shaking at 250 rpm.

### Culture Condition in 50 L Fermenter

Seed culture medium (g/L): YP 10, glucose 40, KH_2_PO_4_ 2, sodium glutamate 5, antifoam agent 0.02, pH 6.0.
Fermentation medium (g/L): YP 15, glucose 50, KH_2_PO_4_ 4, sodium glutamate 5, antifoam agent 0.02, pH 6.8.

5 mL spore solution (10^8^/mL) was inoculated into 1,000 mL baffled flask containing 200 mL seed culture medium and cultivated at 28°C for 48 h under constant orbital shaking at 120 rpm. The seed culture was transferred into 50 L fermenter containing 28 L of the seed medium and cultivated at 28°C for 48 h. Finally, 6 L of the seed culture was inoculated in the 50 L fermenter (Baoxing bioengineering equipment, China) containing 24 L of the fermentation medium.

As for the seed culture, dissolved oxygen concentration was controlled at 20–40% by adjusting air flow rate and agitation speed, while pH was not controlled. As for the fermentation in 50 L fermenters, dissolved oxygen concentration was controlled over 50% of air saturation by adjusting aeration and agitation speed. The aeration rate was controlled 2–3 VVM (airflow/fermentation volume, L/L/min, standard temperature, and pressure) while the agitation speed was controlled 250–400 rpm according to the requirement of dissolved oxygen. During cultivation, glucose solution (500 g/L stock solution) was continuously fed to the fermentation broth to maintain the glucose concentration at a range of 5–15 g/L. YE (N content: 8.0%, solids 78%, Brewer’s yeast extract, Angel Yeast Co., Ltd., China) (450 g/L) was fed to control different RQ. 100 mL of the fermentation broth was taken periodically for examination. Different fed-batch operations were described as follow:

Control: RQ was not controlled.
S1: Strategy1-RQ was controlled at 1.5 from 11 to 160 h.
S2: Strategy2-RQ was controlled at 1.2 from 11 to 160 h.
S3: Strategy3-RQ was controlled at 1.1 during 11–48 h and 1.5 during 48–160 h.

### Culture Condition in 200 m^3^ Fermenter

Seed culture medium (g/L): YP 10, glucose 40, KH_2_PO_4_ 2, sodium glutamate 5, antifoam agent 0.02, pH 6.0.
Fermentation medium (g/L): YP 15, glucose 50, KH_2_PO_4_ 4, sodium glutamate 5, antifoam agent 0.02, pH 6.8.

5 mL spore solution (10^8^/mL) was inoculated into 1,000 mL baffled flask containing 200 mL seed medium and cultivated at 28°C for 48 h under constant orbital shaking at 120 rpm. The seed culture was transferred into 100 L fermenter containing 60 L of the seed culture medium and cultivated at 28°C for 48 h. 60 L of the seed culture was inoculated into the 3 and 30 m^3^ sequentially for scale-up and finally 33 m^3^ of seed broth was transferred into the 120 m^3^ volume of fermentation medium.

As for the seed culture, dissolved oxygen concentration was controlled at 20–40% by adjusting aeration rate. As for the fermentation in 200 m^3^ airlift fermenter, dissolved oxygen concentration was controlled over 50% of air saturation by adjusting aeration. The location of the probe was located at the middle of the fermenter, and the aeration rate was controlled 0.5–1 VVM according to the requirement of dissolved oxygen. During cultivation, glucose solution (800 g/L stock solution) was continuously fed to the fermentation broth to maintain the glucose concentration at a range of 5–15 g/L. YE solution (450 g/L) was fed according to the Strategy3 (S3).

### Sampling and Analytical Methods

#### Determination of Biomass, Residual Sugar, and Amino Nitrogen Concentration

To determine biomass, 100 mL fermentation broth was filtered by medical gauze and dried at 105°C to a constant weight. The residual glucose concentration was measured by using a glucose analyzer (SBA-40C, Institute of Biology, Shandong Academy of Sciences, China) and amino nitrogen concentration was measured according to the method reported by Chen et al. (2008).

#### Determination of Lipid and ARA Content

Dry biomass was broken into powder by a grinder and 5.0 g dry biomass was accurately measured and then transferred into a Soxhlet extractor with 30 mL anhydrous diethyl and extracted for 2 h at 65°C, the solvent was removed in a rotary evaporator. Lipid was then measured by weighing the residue.

The lipid was accurately weighed and then transferred into a 25 mL measuring flask with a volume of 5 mL 1% internal standard tritridecanoin solution and 5 mL 0.5 mol/L NaOH/ethanol, followed by adding 5 mL 33% BF_3_, 5 mL saturated NaCl solution, each procedures were kept at 60°C for 5 min and then mixed evenly. Finally, 5 mL n-hexane was added to the sample followed by mixing thoroughly for 3 min. An 0.5 mL aliquot of the sample was transferred to the centrifuge tube and centrifuged at 5,000 × *g* for 5 min. 1 µL supernatant was then took into the gas chromatograph (Agilent Tech 7890B, USA) equipped with a flame ionization detector and a capillary column DB-23 (30 m × 250 μm × 0.25 μm). The injector and detector temperature was 300°C. The temperature program was defined as follows: initial temperature 90°C for 1 min, increasing to 240°C (ramp rate 9°C/min), stand-by for 5 min, increasing to a final temperature of 250°C (ramp rate 3°C/min). Nitrogen was used as the carrier gas at 1.33 mL/min. Hydrogen and air were used at speeds of 30 and 200 mL/min, respectively. ARA content in the lipid was quantified based on its peak area relative to the standard.

#### Determination of CER, OUR, and RQ

Calculation of the OUR (oxygen uptake rate, mmol/L.h), CER (carbon dioxide evolution rate, mmol/L.h), and RQ values from the O_2,out_ (the oxygen concentration in the outlet gas from the fermenter), CO_2,out_ (the carbon dioxide concentration in the outlet gas from the fermenter), and VVM_in_ (airflow rate/fermentation volume, L/L/min, standard temperature and pressure) data were according to the following equations. O_2,out_, CO_2,out_ were analyzed by Gas analysis equipment (PS7000 German H&B). Thermal mass flowmeter was equipped in 50 L fermenter and vortex shedding flowmeter was used in 200 m^3^ fermenter to measure the airflow rate, both of which were adjusted to the standard temperature and pressure (273.15 K, 101 kPa):

OUR=(20.9%−O2,out∗(79.1%1−O2,out−CO2 ,out)) ∗VVMin∗1000∗60/22.4

CER=(CO2,out∗(79.1%1−O2,out−CO2,out)−0.03%)∗VVMin∗1000∗60/22.4

$$\text{RQ}=\frac{\text{CER}}{\text{OUR}}.$$

## Results and Discussion

### Fed-Batch Fermentation for ARA Production by *M. alpina* in 50 L Fermenter

**Figure 1:**
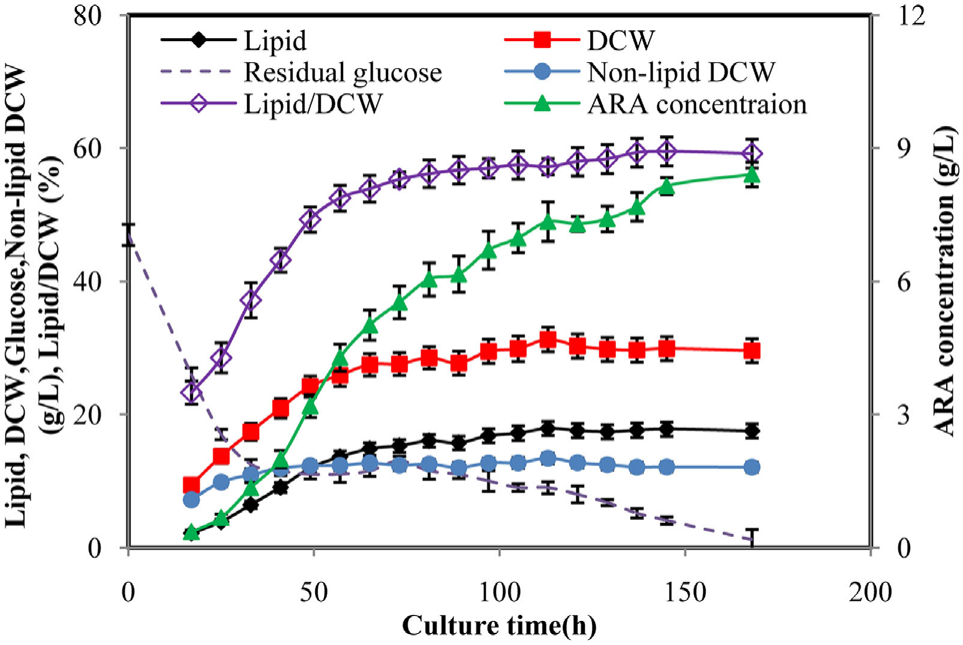
Time courses of arachidonic acid (ARA), lipid, dry cell weight (DCW), glucose concentration in fed-batch fermentation by *Mortierella alpina*. Glucose solution (500 g/L) was continuously fed to the fermentation broth to maintain the glucose concentration at a range of 5–15 g/L. Dissolved oxygen was controlled higher than 50% by adjusting air flow rate and agitation speed.

Time courses of typical cell growth, glucose consumption, lipid synthesis, and ARA production of *M. alpina* during fed-batch cultivation were shown in Figure 1. During the fed-batch fermentation process, glucose solution (500 g/L) was fed to keep the glucose concentration between 5 and 15 g/L after the initial glucose was lower than 15 g/L. Glucose (47 g/L) was quickly consumed in the first 32 h and biomass was increased rapidly accordingly for the first 65 h and then more slowly attained 31.28 g/L at 113 h, which was the maximal. Cell growth was stopped at 113 h until the end of the cultivation. The lipid synthesis was rapidly increased in parallel with the cell growth to achieve 14.8 g/L for the first 65 h, after that it was increased much more slowly from 14.8 to 17.89 g/L and then stopped. Growth-coupled lipid production had been observed in the previous studies for *M. alpina* (Hwang et al., 2005; Wu et al., 2017). The ARA titer was increased during the whole cultivation with a final concentration of 8.4 g/L, showing productivity of 1.2 g/L per day. In the fed-batch fermentation, lipid/dry cell weight (DCW) reached 59.2%, which was much higher than most of the oleaginous fungus (Ling et al., 2016; Wu et al., 2017). Obviously, low concentration of biomass was a limitation for lipid synthesis because lipid was stored within the existing mycelium, which could no longer divide or elongate. Thus, high concentration of biomass was essential for ARA-rich oil production.

### Screening of Organic Nitrogen in Shake Flask Culture

Considering that the carbon source was continuously fed into the fermentation broth, addition of extra nitrogen source would be necessary for increasing the cell growth. According to the study by Lu et al. (2011), organic nitrogen was favorable for both cell growth and lipid synthesis. To increase the biomass and ARA production of *M. alpina*, addition of different organic nitrogen sources were performed in shake flask cultivation.

As showed in Table 1, YP showed no promotion for cell growth. This might be due to that YP was insoluble nitrogen source, which was not so easy for the strain to absorb. As the YP concentration increased, lipid/DCW was slightly lowered but the ARA/lipid, as a fraction of total lipids, was slightly higher than the control. Soybean cake (SC) gave the maximal biomass (52.1 g/L) but the lowest ARA/lipid (no more than 24%), which resulted in the lowest ARA titer. When corn steep liquor (CSL) was supplemented at a final concentration of 1%, ARA titer reached 7.43 g/L, which was 1.14 times of the control. However, addition of CSL showed no benefit for the cell growth. The highest ARA titer (7.78 g/L) was showed in the culture by adding 0.5% YE, which was 1.19 times of the control. DCW and lipid production also increased by 33.7 and 19.8% compared to the control, respectively. As the concentration of YE increased, lipid/DCW and ARA/lipid were decreased. That was because lipid accumulation was limited when nitrogen was sufficient (Jin et al., 2007). In all cases, addition of YE was best for ARA production although the medium containing SC was favorable for cell growth and lipid production. Jang et al. (2005) also found that YE could enhance cell growth and total lipid accumulation by *M. alpina*. However, some of previous works considered that YE was not suitable for large-scale industrial ARA production because of its high cost (Lu et al., 2011). Nevertheless, since ARA rich oil is a commercial product used widely in the infant formula, the safety and stableness of the organic nitrogen are more important than the price, besides, the total cost of the nitrogen source and the final yield of ARA should also be taken into consideration. YE is the end product from the autolysis process of commercial yeast cells by combined enzymatic treatments (Milic et al., 2007). Thus, YE production is more stable and reliable than the plant source nitrogen in the way of output, quality, and food safety.

**Table 1:** Effects of nitrogen on parameters of dry cell weight (DCW), lipid, arachidonic acid (ARA), lipid/DCW, and ARA/lipid in shake flask fermentations by *Mortierella alpina*.

	Concentration (%)	DCW (g/L)	Lipid (g/L)	ARA (g/L)	Lipid/DCW (%)	ARA/lipid (%)
Control	\	29.80 ± 0.40	13.89 ± 0.39	6.53 ± 0.36	46.61 ± 0.69	47.04 ± 1.29
Yeast powder	0.5	32.10 ± 0.21	14.28 ± 0.40	6.67 ± 0.32	44.50 ± 0.95	46.70 ± 0.96
	1	33.20 ± 3.03	14.20 ± 0.49	6.84 ± 0.03	42.76 ± 2.68	48.20 ± 1.92
	1.5	32.57 ± 1.49	13.58 ± 0.42	6.59 ± 0.09	41.7 ± 0.65	48.50 ± 0.83
Yeast extract	0.5	39.84 ± 0.34	16.64 ± 0.01	7.78 ± 0.17	41.76 ± 0.39	46.75 ± 1.01
	1	43.16 ± 0.99	14.80 ± 0.49	6.35 ± 0.37	34.30 ± 0.36	42.86 ± 1.10
	1.5	46.53 ± 1.16	14.35 ± 0.16	5.80 ± 0.14	30.85 ± 0.43	40.39 ± 1.48
Corn steep liquor	0.5	30.23 ± 1.24	14.30 ± 0.11	6.97 ± 0.02	47.32 ± 2.20	48.72 ± 0.49
	1	32.52 ± 0.66	15.05 ± 0.10	7.43 ± 0.14	46.27 ± 0.63	49.37 ± 1.21
	1.5	30.15 ± 2.33	14.63 ± 0.61	7.11 ± 0.10	48.52 ± 1.61	48.63 ± 1.30
Soybean cake	0.5	38.40 ± 0.44	16.36 ± 0.25	3.81 ± 0.18	42.60 ± 1.14	23.30 ± 1.42
	1	47.30 ± 1.11	21.14 ± 0.09	3.95 ± 0.13	44.70 ± 0.83	18.70 ± 0.51
	1.5	52.10 ± 0.62	23.81 ± 0.04	3.24 ± 0.41	45.70 ± 0.48	13.60 ± 1.73

### Effect of YE Feeding on the Changes of RQ

**Figure 2:**
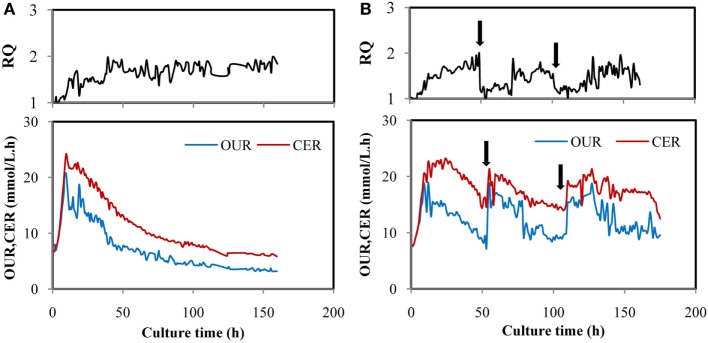
Time courses of OUR, CER, and respiratory quotient (RQ) in fed-batch fermentation by *Mortierella alpina* in control **(A)** and trial batches feeding yeast extract (YE) **(B)**. YE solution (450 g/L) was fed intermittently at 50 and 100 h and the YE concentration in the fermentation broth reached 5 g/L for each time. In all trials, glucose solution (500 g/L) was continuously fed to the fermentation broth to maintain the glucose at a range of 5–15 g/L. Dissolved oxygen was controlled higher than 50% by adjusting air flow rate and agitation speed.

In order to learn the effect of YE feeding on the change of RQ, YE solution (450 g/L) was fed at 50 h to reach a final concentration of 5 g/L in the fermentation broth, and the same amount of YE was fed at 100 h. Figure 2A showed time courses of RQ, OUR, and CER in a fed-batch fermentation of *M. alpina* without feeding YE. RQ kept at 1.0 before 10 h and then increased to 1.8, after that, RQ was maintained at a dynamic equilibrium till the end of the fermentation. OUR and CER increased exponentially in the first 10 h and then decreased very quickly from 10 to 100 h, after that, OUR maintained around 4.27 mmol/L.h and CER maintained around 6.37 mmol/L.h. The possible explanation for RQ changes was that metabolic flux of glucose differed in different stages. Theoretically, with sufficient supply of oxygen and nitrogen, glucose would be channeled into the citric acid cycle (TCA cycle) to provide the energy source for cell growth, RQ was approximately 1.0 based on the reaction of C_6_H_12_O_6_ + 6O_2_ = 6CO_2_ + 6H_2_O, which was verified in many studies (Xiong et al., 2010; Chen et al., 2015). When glucose was utilized for fatty acids synthesis, more CO_2_ would be produced and less O_2_ would be consumed (Ratledge, 2014), which led to a higher RQ of more than 1.0. OUR and CER were calculated by O_2_ and CO_2_ measured by gas analyzer. When more O_2_ was consumed and more CO_2_ was produced, OUR and CER would increase correspondingly while VVM_in_ remained unchanged. CO_2_ were produced in the cell growth *via* TCA cycle and lipid production process. So, the production of CO_2_ was dependent on the synergistic effect of both the two processes. The decrease of OUR and CER might be caused by the cessation of cell growth. Similar results had been presented by Wynn et al. (2001), who found that CO_2_ evolution by *Mucor Circinelloides* reached the peak rapidly before 8 h and then decreased quickly though the lipid was still produced.

Figure 2B showed that when YE was fed to the fermentation broth at 50 h, OUR and CER increased quickly while RQ dropped from 1.8 to 1.2 and then maintained around 1.2 for about 22 h. The same law was showed when YE was supplemented at 100 h. Obviously, cell growth was activated again when YE was fed because OUR and CER increased, meaning that more O_2_ was consumed and more CO_2_ was produced. Furthermore, RQ was reduced to 1.2 from 1.8, indicating that the cell growth might take dominate again (Streekstra, 2010). It was supposed that more O_2_ was consumed and more CO_2_ was produced in the stage of cell growth than the stage of lipid synthesis because OUR and CER increased by one times more when YE was fed. It may also account for the decrease of OUR and CER showed in Figure 2A. The phenomenon indicated that RQ was in a strong connection with YE feeding and may be an ideal online parameter for regulating YE feeding.

### The Comparison of Different YE Feeding Strategies

In order to obtain a deep understanding of effect of YE feeding on cell growth, lipid, and ARA production, different online RQ control strategies were applied in 50 L fermenters. YE feeding strategies were mentioned in the Section “Culture Condition in 50 L Fermenter.”

**Figure 3:**
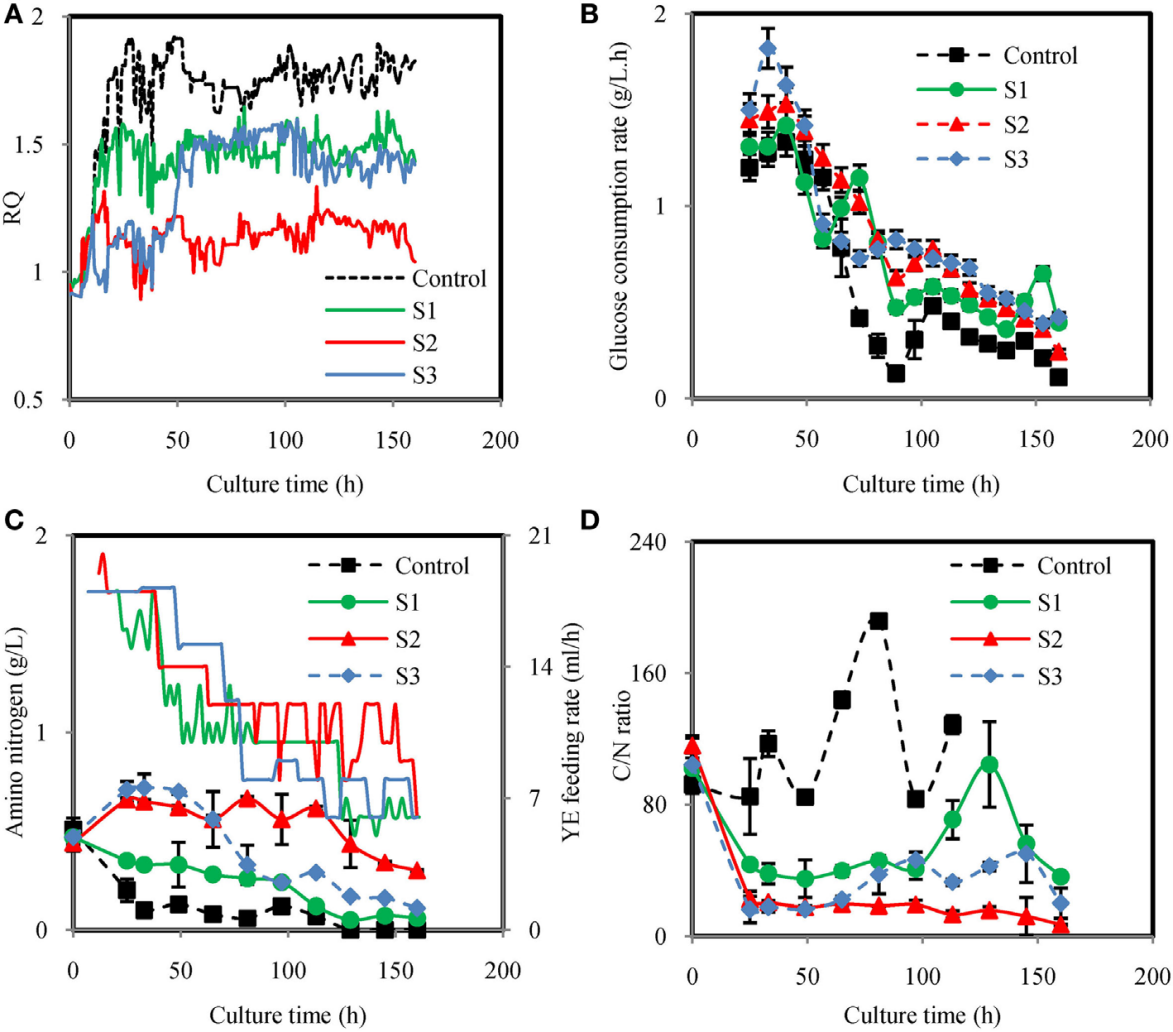
Time courses of respiratory quotient (RQ) **(A)**, glucose consumption rate **(B)**, amino nitrogen concentration and yeast extract (YE) feeding rate **(C)**, C/N ratio **(D)** under different YE feeding strategies. Control: RQ was not controlled; S1: Strategy1-RQ was controlled at 1.5 from 11 h to 160 h; S2: Strategy2-RQ was controlled at 1.2 from 11 h to 160 h; S3: Strategy3-RQ was controlled at 1.1 during 11–48 h and 1.5 during 48–160 h.

Figure 3 showed the time courses of the fermentation parameters under different feeding strategies. Under different YE feeding strategies, RQ were effectively controlled at set values (Figure 3A). The glucose consumption rates were not significantly different in the YE feeding cultures but higher than the control from 73 h to the end of fermentation (Figure 3B). The total amount of glucose consumed in Control, S1, S2, and S3 were 81.20, 112.18, 123.56, and 130.00 g/L, respectively (Table 2). Figure 3C showed the YE feeding rate and the amino nitrogen concentration in the fermentation broth. The amino nitrogen concentration was significantly different from each other. The highest concentration was observed in S2 while the lowest amino nitrogen was observed in the control. These results indicated that feeding YE could effectively affect RQ and enhance the consumption of glucose. Figure 3D showed the carbon to nitrogen ratio (C/N ratio) in the four different cultures. The C/N ratio was calculated based on the residual glucose and amino nitrogen concentration. As showed in Figure 3D, the C/N ratio in the control was the highest while the lowest C/N ratio was observed in S2. It was confusing that the C/N ratio in the control was much higher than in the other cultures. In order to figure it out, the total C/N ratio was calculated based on the total amount of glucose and nitrogen source consumed in the cultures. The total C/N ratios in the four cultures were 24.26, 20.03, 19.21, and 20.18, respectively (Table 2). By comparison with all these C/N ratios, it could be inferred that the insoluble nitrogen source YP may exert a major effect on the C/N ratio in the culture process because YP would slowly release the amino nitrogen. Still, the C/N ratio gave us the instant information of the medium during the cultivation. Since the residual glucose in the fermentation process was controlled based on the same strategy, the C/N ratio in the process was mainly decided by the nitrogen feeding. Thus, feeding YE based on RQ might also control the C/N ratio, which actually controlled the division of carbon flux between cell growth and lipid synthesis. Yield coefficient of non-lipid DCW to N-nitrogen (*Y*_X/N_) for S2 was the highest (Table 2), indicating that YE was highly used for biomass synthesis. *Y*_X/N_ for control was the lowest meaning that the biomass was not efficiently produced. It coincided with the results showed in Table 2 that S2 attained the highest non-lipid DCW while the control attained the lowest non-lipid DCW.

**Table 2:** Various yields in fermentations using different fermentation strategies in the lab and industrial scale fermenters.

Parameters	Different fermentation methods in lab and industrial scale fermenters					
	50 L-control	50 L-S1	50 L-S2	50 L-S3	200 m^3^-control	200 m^3^-S3
Dry cell weight (DCW) (g/L)	29.60	43.70	57.68	53.10	39.55	63.99
Non-lipid DCW	12.08	21.50	37.20	26.44	16.22	30.82
Yeast extract feeding (g/L)	0.00	11.27	15.42	15.47	0.00	18.90
Glucose (g/L)	81.20	112.18	123.56	130.00	108.45	168.20
Total C/N ratio (g/g)	24.26	20.03	19.21	20.18	27.29	21.69
Lipid (g/L)	17.51	22.20	20.48	26.66	23.33	33.17
Arachidonic acid (ARA)/lipid (%)	48.00	43.50	31.50	43.10	51.12	48.23
ARA (g/L)	8.41	9.66	6.45	11.49	11.93	16.82
ARA productivity (g/L/day)	1.20	1.38	0.92	1.64	1.70	2.40
*Y*_ARA/S_ (g/g)	0.10	0.09	0.05	0.09	0.11	0.10
*Y*_ARA/DCW_ (g/g)	0.28	0.22	0.11	0.22	0.30	0.26
*Y*_Lipid/S_ (g/g)	0.22	0.20	0.17	0.21	0.22	0.20
*Y*_X/S_ (g/g)	0.15	0.19	0.30	0.20	0.15	0.18
*Y*_X/N_ (g/g)	9.02	9.60	14.46	10.26	10.20	9.94

*Total C/N ratio, the carbon to nitrogen ratio based on the total amount of glucose and nitrogen source*.

*Y_ARA/S_, conversion of glucose to ARA; Y_ARA/DCW_, yield coefficient of ARA to DCW; Y_Lipid/S_, conversion of glucose to lipid; Y_X/S_, conversion of glucose to non-lipid DCW; Y_X/N_, yield coefficient of non-lipid DCW to N-nitrogen*.

**Figure 4:**
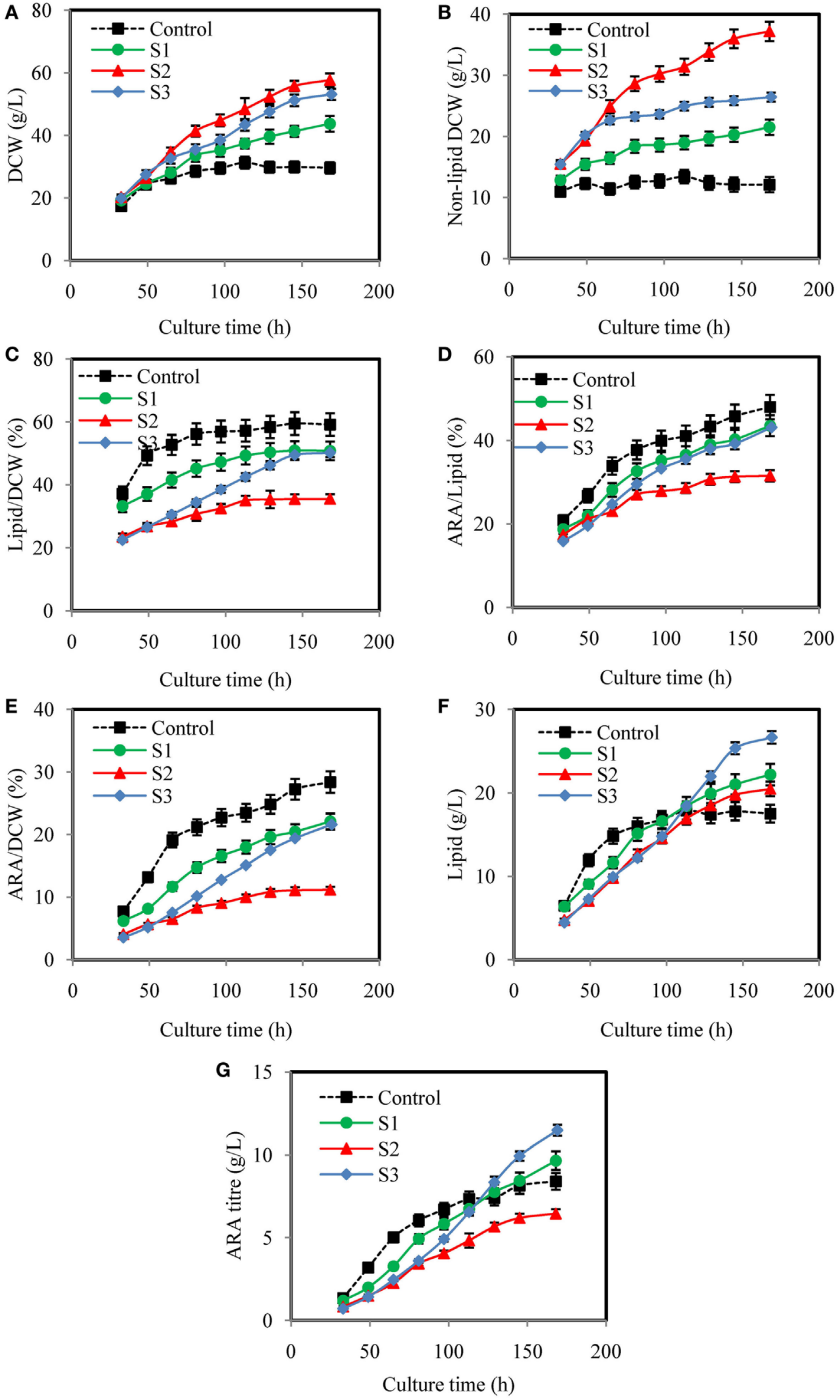
Effects of different yeast extract (YE) feeding strategies on dry cell weight (DCW) **(A)**, non-lipid DCW **(B)**, lipid/DCW **(C)**, arachidonic acid (ARA)/lipid **(D)**, ARA/DCW **(E)**, lipid **(F)**, and ARA titer **(G)**, in submerged fermentations of *Mortierella alpina* in 50 L fermenter. Glucose solution (500 g/L) was continuously fed to the fermentation broth to maintain glucose concentration at a range of 5–15 g/L. YE solution of 450 g/L was fed to the fermentation broth according to different strategies (S1, S2, and S3). Dissolved oxygen was controlled higher than 50% by adjusting air flow rate and agitation speed. Control: respiratory quotient (RQ) was not controlled; S1: Strategy1-RQ was controlled at 1.5 from 11 h to 160 h; S2: Strategy2-RQ was controlled at 1.2 from 11 h to 160 h; S3: Strategy3-RQ was controlled at 1.1 during 11–48 h and 1.5 during 48–160 h.

Figure 4 showed dynamic courses of DCW, non-lipid DCW, lipid/DCW, ARA/lipid, lipid, and ARA concentration using different YE feeding strategies. As showed in Figure 4A, feeding YE efficiently increased DCW. In the culture of S1, S2, and S3, DCW kept increasing in a linear manner with different slopes, which were markedly higher than Control. Figure 4B showed that the maximal non-lipid DCW in culture of S1 and S3 were 21.5, 26.4 g/L, which were 74.4 and 40.9% lower than S2, but were 78% and 119% higher than the control, respectively. Non-lipid dry cell weight (non-lipid DCW) was an important parameter, which indicated the cell growth of oleaginous microorganisms because it excluded the effect of lipid synthesized in the cell. It was observed that RQ in S2 was the lowest in all the culture while RQ in control was the highest (Figure 3A). Thus, it was concluded that when RQ was controlled lower by feeding YE, the amino nitrogen concentration would be higher, thus the cell growth rate would be higher than the control. Guo et al. (2016) also found that when RQ was controlled at 1.2 by enhancing the aeration rate to 1.5 VVM in DHA production by *Schizochytrium* sp., the biomass was the higher than the batches, which RQ was controlled at 1.7 or 2.0. So, RQ was a good online parameter to indicate the cell growth of oleaginous microbes.

Although feeding YE could enhance the cell growth, how it was fed significantly affected the lipid/DCW and ARA/lipid. As showed in Figures 4C–E, the highest lipid/DCW (59.18%), ARA/lipid (48.0%), and ARA/DCW (28%) were observed in the control and the lowest lipid/DCW (35.50%), ARA/lipid (31.5%), and ARA/DCW (11%) were obtained in S2. Combined with the analysis of RQ changes showed in Figure 3A, it could be inferred that RQ was also highly relevant with ARA/lipid, lipid/DCW, and ARA/DCW. As previously observed, the synthesis of fatty acids was dependent on the provision of acetyl-CoA and NADPH (Ratledge, 2004), and both the reactions of acetyl-CoA and NADPH formation were accompanied by CO_2_ emission and no O_2_ consumed (Wynn and Ratledge, 2000; Wynn et al., 2001; Zhang et al., 2007; Belle and Goossens, 2011). So, the possible reason for the decrease in the ARA/lipid, lipid/DCW, and ARA/DCW with lower RQ was that less glucose was channeled to the lipid synthesis and more was channeled to the cell growth. One proof was showed in Table 2 that conversion of glucose to ARA (*Y*_ARA/S_) and lipid (*Y*_Lipid/S_) in the control (RQ ≈ 1.8) were the highest while conversion of glucose to non-lipid DCW (*Y*_X/S_) was the lowest. In the culture of S2, opposite results were showed. Guo et al. (2016) also found that when RQ was controlled lower, the DHA/lipid was lower in the cultivation of *Schizochytrium* sp. for production of docosahexaenoic acid. Another explanation for the difference of ARA/lipid and lipid/DCW could be made in the sight of amino nitrogen in the fermentation broth. As shown in Figure 3C, the amino nitrogen in the control was rapidly consumed in the first 33 h and then maintained the lowest concentration during the whole fermentation process while the amino nitrogen concentration in the S2 was the highest due to YE feeding method. With sufficient nitrogen and carbon, the cell growth was greatly enhanced and lipid accumulation was limited. The result was in accordance with report by Jin et al. (2007) that higher nitrogen concentration generally resulted in more biomass and lower lipid and ARA content.

It was not satisfied that the strategies for feeding YE could not enhance the lipid/DCW and ARA/lipid, which was consistent with the results from the shake flask cultures (Table 1). However, industrial ARA production focused mainly on the final concentration of lipid and ARA. In this respect, the strategy S2 and S3 showed satisfied results on lipid and ARA production. As shown in Figure 4F, the lipid production in the control were the fastest in all cases before 65 h, but slowed down after 65 h till the end of cultivation. However, the lipid production in the culture of S1, S2, and S3 kept increasing continually and the maximal titer reached 22.2, 20.48, and 26.66 g/L, respectively, whereas only 17.51 g/L lipid was produced in the corresponding control experiment. Obviously, S3 could improve lipid production by 52.2%, which was the highest (Figure 4F). The ARA titer in S3 reached 11.49 g/L, which was also the highest in all cultures (Figure 4G). The reason why S3 could enhance lipid and ARA production significantly might due to the two stage control of RQ. When RQ was controlled at 1.1 before 48 h, more YE solution was fed to the fermentation broth and the cell growth was greatly promoted (Figure 4B). However when RQ was set at 1.5 after 48 h, cell growth slowed down because amino nitrogen concentration was lower due to the YE feeding speed slowing down. Lipid accumulation was observed to accelerate because lipid/DCW was increasing rapidly after 48 h (Figure 4C). This was in accordance with the study by Wu et al. (2017), who divided the ARA production process into four stages, one of which was for cell growth and one for lipid accumulation. However, in the culture of S3, YE solution was continually fed to the fermentation broth during the lipid accumulation stage in which an N-limitation condition was considered to be needed according to many studies (Belle and Goossens, 2011; Laoteng et al., 2011). It seemed to be contradicted with traditional theory in this study. However, Wynn et al. (1999) found that the malic enzyme would be activated again by feeding some nitrogen in the lipid accumulation stage, which was important for removing NADPH limitation and thus enhanced the lipid synthesis. It might explain the reason why feeding YE at appropriate speed would enhance lipid accumulation in our study. The C/N ratio might explain the different results of the four cultures in another way. Feeding YE lowered the total C/N ratio from 24.26 to an average of 19.81 (Table 2). Park et al. (2001) found that the optimal total C/N ratio was in the range of 15–20 in terms of ARA oil accumulation, which was in accordance with this study. However, the instant C/N ratio in the process differed in different stages. The optimal strategy S3 maintained lower C/N ratio in the first stage (25–49 h) and then kept higher C/N ratio in the second stage (65–145 h). It was inferred that lower C/N ratio would enhance cell growth while higher C/N ratio promote lipid synthesis, which was verified in many studies (Stredanská and Šajbidor, 1993; Braunwald et al., 2013). The S2 culture maintained a C/N ratio at approximately 20 after 25 h, which attained much higher non-lipid DCW. So, the RQ actually controlled the C/N ratio in the culture and resulted in the division of carbon flux between cell growth and lipid synthesis.

Although the lipid production obtained from the culture of S2 was higher than the control, ARA titer was lower than the control due to the lower ARA/lipid. It was observed that the total amount of YE used in S2 and S3 was very close (showed in Table 2), but the titers of lipid and ARA were totally different. S3 was significantly better in lipid and ARA production than S2. By comparing the amount of YE used in the process, it was found that more YE was fed before 60 h in S3 (Figure 3C). However, feeding more YE at the beginning or loading all YE in the initial medium without the feeding of YE in the culture process would not be a better choice because the lipid and ARA production were lowered as the YE amount increased in the initial medium (Table 1). It was inferred that YE significantly affected the C/N ratio, which was an important parameter for lipid production. Park et al. (2001) found that ARA and lipid titers were increased with the C/N ratio increased from 5 to 15, when C/N ratio was higher than 15, both ARA and lipid titers were decreased. Thus, feeding YE to maintain an appropriate C/N ratio was crucial for ARA production. So, the YE feeding strategy was important for ARA production. With RQ as a real-time parameter, the YE feeding speed was adjusted without any delay and a good balance was reached between the cell growth and lipid synthesis.

### Scale-up of YE RQ-Feedback Control to 200 m^3^

Based on the results from 50 L fermenters, the optimal strategy S3 was applied to 200 m^3^ fermenter. Table 2 listed results from the control batches and batches using the online RQ-feedback strategy S3 (the detailed data were shown in supporting materials). DCW, lipid, and ARA titer were 63.99, 33.17, and 16.82 g/L, which were 1.68, 1.42, and 1.41 times of the control, respectively. However, the ARA/lipid (48.23%) was lower than the control (51.12%), which was in accordance with the result from the lab-scale trials. The yield coefficients of *Y*_ARA/S_, *Y*_Lipid/S_, and *Y*_X/S_ in the cultures of 200 m^3^ fermenter were not significantly different with the cultures in 50 L fermenter. The yield coefficient of ARA to DCW (*Y*_ARA/DCW_) in the control and 200 m^3^-S3 were 0.3 and 0.26 g/g, respectively. Obviously the optimized process using S3 strategy got lower *Y*_ARA/DCW_, which would increase the downstream processing cost. In addition, the optimized process consumed extra amount of 18.9 g/L YE and 59.75 g/L glucose and got 41% more net ARA than the control. The comprehensive yield of ARA and the costs for each batch were calculated. As a result, the cost per unit product (cost/1 kg ARA) decreased by 11.1% compared with the control. Normally, in commercial production of ARA, higher lipid and ARA content in biomass may be desired to reduce the downstream processing cost. Thus, how to increase the ARA titer without decrease of *Y*_ARA/DCW_ is an important point for further study.

An interesting result was observed in the scale-up process. The ARA titers in the cultures of control and S3 in 200 m^3^ fermenter were 11.93 and 16.82 g/L, which were 42 and 50.4% higher than the control and S3 in 50 L fermenter. It may be the more concentrated glucose feed solution was used (800 g/L) in 200 m^3^ fermenter, while the relatively lower concentrated glucose solution was used (500 g/L) in 50 L fermenter. Because strong glucose feed would cause less dilution effect (less feed volume into the fermenter caused less dilution and then resulted in higher concentrations of DCW and ARA titer). This could explain very well for why the 200 m^3^ achieved much higher biomass and ARA titer than the 50 L fermenter.

Table 3 showed the comparative results of ARA production among previous studies by *M. alpina*. Various studies had been reported to achieve high ARA titer; however, the highest ARA productivity of 2.4 g/L/day was achieved by online RQ-feedback strategy of feeding YE in a 200 m^3^ fermenter, which was the largest scale that had been reported. In conclusion, RQ feedback strategy for YE feeding was successfully realized in the 200 m^3^ fermenter. Thus, RQ could be an effective online parameter for scale-up of ARA production and could also be applied to other industrial production of microbial oil by oleaginous microorganisms.

**Table 3:** Comparative results of arachidonic acid (ARA) production in previous studies by *Mortierella alpina*.

Microorganism	ARA yield/culture time	ARA productivity (g/L/day)	Scale	Reference
*M. alpina ME-1*	15.9 g/L/7 days	2.27	5 L	Peng et al. (2010a,b)
*M. alpina ME-1*	19.8 g/L/11 days	1.80	5 L	Jin et al. (2008)
*M. alpina ME-1*	19.02 g/L/12 days	1.59	5 L	Jin et al. (2009)
*M. alpina DSA-12*	18.8 g/L/12.5 days	1.50	13 L	Hwang et al. (2005)
*M. alpina R807*	13.5 g/L/7.5 days	1.81	7.5 L	Wu et al. (2017)
*M. alpina LPM 301*	10 g/L/7 days	1.43	250-mL flask	Zeng et al. (2012)
*M. alpina 1* *S-4*	10.9 g/L/8 days	1.36	10 kL	Higashiyama et al. (1998a,b)
*M. alpina ME-1*	9.2 g/L/7 days	1.31	5 L	Peng et al. (2010a,b)
*M. alpina 1 S-4*	13 g/L/10 days	1.30	10 kL	Higashiyama et al. (1998a,b)
*M. alpina ATCC 32222*	11 g/L/11 days	1.00	250-mL flask	Singh and Ward (1997)
*M. alpina I49-N18*	11.49 g/L/7 days	1.64	50 L	This study
*M. alpina I49-N18*	16.82 g/L/7 days	2.40	200 kL	This study

## Conclusion

In present work, an online RQ-feedback strategy of feeding YE for efficient ARA production was developed and then scaled up to the 200 m^3^ fermenter for the first time during the submerged cultivation of *M. alpina* I49-N18. YE was proved to be the best organic nitrogen, which could efficiently increase cell growth in shake flask culture. By feeding YE to control RQ at 1.1 during 0–48 h and at 1.5 during 48–160 h, DCW and ARA titers reached 53.1 and 11.5 g/L in 50 L fermenter, which were increased by 79.4 and 36.9% as compared to that without YE feeding, respectively. An average ARA production of 16.82 g/L was obtained from 12 continuous batches in 200 m^3^ bioreactor fermentation by using the online RQ-feedback strategy, which was 41.0% higher than the control batches. Based on these results, it can be concluded that the online RQ-feedback strategy of feeding YE was proved to be efficient for ARA production.

## Author Contributions

XL and CY have carried out the experiments and the writing of the manuscript. JY and ZW have managed and supervised the work throughout all and they are accountable for all aspects of the work. SL has designed this work.

## Conflict of Interest Statement

Authors XL, CY, JY, ZW, and SL were employed by company CABIO Biotechnology (Wuhan) Co., Ltd. All other authors declare no competing interests.
