# Supplementary material for: An Online Respiratory Quotient-Feedback Strategy of Feeding Yeast Extract for Efficient Arachidonic Acid Production by Mortierella alpina
Source: Front Bioeng Biotechnol. 2018 Jan 22;5:83. doi: 10.3389/fbioe.2017.00083 (PMC5786879; doi:10.3389/fbioe.2017.00083)
Supplement: Supplementary file 1 [file Table_1.DOCX]

**Supporting Information**

Table 1 Yields of ARA in fermentations in 200m^3^ fermenter

| Batch | Culture time(h) | Biomass(g/L) | Lipid (g/L) | ARA/ lipid (%) | ARA (g/L) |
| --- | --- | --- | --- | --- | --- |
| Control-1 | 168 | 39.98 | 24.11 | 50.95 | 12.28 |
| Control-2 | 168 | 40.12 | 23.69 | 51.10 | 12.10 |
| Control-3 | 168 | 38.55 | 22.20 | 51.30 | 11.39 |
| S3-1 | 168 | 64.12 | 34.78 | 47.80 | 17.50 |
| S3-2 | 168 | 63.67 | 32.30 | 48.20 | 16.39 |
| S3-3 | 168 | 62.45 | 31.92 | 48.80 | 16.40 |
| S3-4 | 168 | 65.08 | 33.08 | 46.90 | 16.33 |
| S3-5 | 168 | 63.46 | 33.46 | 48.10 | 16.94 |
| S3-6 | 168 | 65.35 | 32.28 | 47.90 | 16.28 |
| S3-7 | 168 | 64.12 | 35.06 | 48.80 | 17.52 |
| S3-8 | 168 | 65.09 | 34.44 | 48.10 | 17.44 |
| S3-9 | 168 | 62.74 | 31.89 | 49.30 | 16.76 |
| S3-10 | 168 | 64.63 | 32.79 | 47.10 | 16.26 |
| S3-11 | 168 | 63.02 | 33.11 | 48.67 | 16.96 |
| S3-12 | 168 | 64.17 | 32.98 | 49.12 | 17.05 |
| Control-Average | 168 | 39.55±0.87 | 23.33±1.00 | 51.12±0.18 | 11.93±0.47 |
| S3-Average | 168 | 63.99±0.95 | 33.17±1.08 | 48.23±0.75 | 16.82±0.49 |
